# Effect-based assessment of recipient waters impacted by on-site, small scale, and large scale waste water treatment facilities – combining passive sampling with *in vitro* bioassays and chemical analysis

**DOI:** 10.1038/s41598-018-35533-x

**Published:** 2018-11-21

**Authors:** Anna Kjerstine Rosenmai, Johan Lundqvist, Pablo Gago-Ferrero, Geeta Mandava, Lutz Ahrens, Karin Wiberg, Agneta Oskarsson

**Affiliations:** 1 Department of Biomedical Sciences and Veterinary Public Health, Swedish University of Agricultural Sciences, Box 7028, SE-750 07 Uppsala, Sweden; 2 Department of Aquatic Sciences and Assessment, Swedish University of Agricultural Sciences, Box 7050, SE-750 07 Uppsala, Sweden

## Abstract

Waste water treatment facilities are a major sources of organic micropollutants (MPs) in surface water. In this study, surface water samples were collected from seven sites along a river system in Uppsala, Sweden, during four seasons and evaluated based on the occurrence of MPs in the samples and bioactivity using *in vitro* bioassays. The sampling sites were differentially impacted by on-site sewage treatment facilities (OSSFs), small scale, and large scale waste water treatment plants (WWTPs). The bioassays used included activation of aryl hydrocarbon receptor (AhR), estrogen receptor (ER), nuclear factor kappa-light-chain-enhancer of activated B cells (NFkB), nuclear factor erythroid 2-related factor 2 (Nrf2), and androgen receptor (AR). Occurrence of 80 MPs, were analyzed using liquid chromatography coupled to tandem mass spectrometry. Most water samples induced AhR activity, and all sampling sites showed a similar profile regarding this activity. With the exception of one water sample, we did not detect any NFkB, Nrf2 or AR activity of the water samples. The exception was a sample impacted by OSSFs, which showed an activity in multiple bioassays, but the activity could not be explained by the occurrence of target MPs. The occurrence of MPs showed a spatial trend, with the highest number and amount of MPs detected in the samples collected downstream of the WWTPs, where up to 47 MPs were detected in one single sample. A seasonal variation was observed with highest levels of MPs and highest AhR activities in samples collected in June and September 2015. However, neither the seasonal activity nor the on-site activity could be explained by the measured MPs, suggesting unknown contributory agents in the water.

## Introduction

Surface water can be contaminated with organic micropollutants (MPs), which is an environmental problem of great concern, both for human health and the ecosystem. One major contributor to MP contamination of surface water is waste water treatment facilities^1^. Besides conventional waste water treatment plants (WWTPs), on-site sewage treatment facilities (OSSFs) can have an impact on the aquatic environment since they are usually not designed to remove MPs from wastewater^2–4^. Data on removal efficiencies of MPs in OSSFs is limited and the current knowledge is based only on chemical analysis of MPs, either in laboratory scale systems^5^ or real OSSFs^6–10^. However, chemical analysis has limitations as typically a restricted number of MPs are targeted, thus excluding the vast majority of possible contaminants and potential transformation products as well as the potential of MPs to induce adverse effects.

*In vitro* bioassays for environmental monitoring of MPs is a rapidly expanding field of research^11–20^, which to some extent can address shortcomings of chemical analysis. While the bioassays cannot identify the specific chemical(s) causing the toxic effects in a given sample, a great strength is that they allow assessment of the total toxicity exerted by all MPs present in a sample – regardless if the toxicity is caused by known anthropogenic compounds, unknown anthropogenic compounds, naturally occurring compounds or a combination of these^21–23^. The selection of suitable biological endpoints to assay with *in vitro* bioassays is of critical importance, to determine toxic activities of a broad range of chemicals. A study by Escher *et al*. (2014) tested a large number of bioassays and concluded that the most responsive and relevant endpoints were related to hormone-mediated modes of action (estrogen receptor (ER), androgen receptor (AR), glucocorticoid receptor (GR), and peroxisome proliferator-activated receptor (PPAR) activities), xenobiotic metabolism (aryl hydrocarbon receptor (AhR) and pregnane x receptor (PXR) activities), and reactive modes of action (genotoxicity and oxidative stress)^23^.

In the present study, we assessed the impact of discharge from OSSFs and WWTPs of various sizes on the aquatic environment by integrating chemical and toxicological analysis of surface water samples using passive sampler on a seasonal basis. The samples were analyzed for bioactivity using *in vitro* assays for specific toxicity endpoints; activation of AhR, nuclear factor kappa-light-chain-enhancer of activated B cells (NFkB), nuclear factor erythroid 2-related factor 2 (Nrf2), and ER, as well as AR agonistic and antagonistic activities. Furthermore, the samples were chemically characterized for the presence of more than 80 MPs. Our study showed a seasonal variation in AhR bioactivity of most water samples and amount of MPs in the water samples most heavily impacted by WWTPs. The chemical analysis could, however, not explain the observed bioactivity.

## Material and Methods

### Chemicals and reagents

The occurrence of 80 MPs was evaluated, including 53 pharmaceuticals belonging to different therapeutic groups (*e*.*g*. analgesics, anesthetics, antibiotics, antidepressants, antidiabetics, antiepileptics, antifungals, antihypertensives, antiulcer, beta-blocking agents, benzodiazepines, diuretics and NSAIDs), 14 per- and polyfluoroalkyl substances (PFASs), 7 personal care products; 3 pesticides, 2 illicit drugs, 2 stimulants and one artificial sweetener. Target analytes with its corresponding CAS numbers are summarized in Table SI1, in the Supplementary Information. In addition, 42 internal standards (IS), isotopically labelled, were used for quantification.

The analytical standards used for quantification were purchased from Sigma-Aldrich (Sweden) and of high purity grade (>95%). Isotopically labelled standards (IS) for PFASs, personal care products (PPCPs), pharmaceuticals, and pesticides were purchased from Wellington Laboratories (Canada), Toronto Research Chemicals (Toronto, Canada), Sigma-Aldrich (Sweden), and Teknolab AB (Kungsbacka, Sweden), respectively. Gradient grade acetonitrile, methanol, and ethyl acetate, used for chemical analysis, were purchased from Merck (Darmstadt, Germany). Ammonium formate, 25% ammonia solution, ammonium acetate and formic acid 98% were purchased from Sigma-Aldrich (Sweden).

A Milli-Q Advantage Ultrapure Water purification system (Millipore, Billercia, MA) was used to produce ultrapure water and the water was filtered using a Millipak Express membrane (0.22 μm).

Polar organic chemical integrative sampler (POCIS) were prepared by placing 200 mg HLB bulk sorbent between two polyethersulfone (PES) membranes (EST, St. Joseph, MO, USA), compressed with two stainless steel rings (EST, St. Joseph, MO, USA).

### Water sampling and sample preparation

Water was sampled at seven sites (Table 1) along the Fyris River water system at four different seasons including samples from November 2014, March 2015, June 2015, and September 2015. The sampling sites are differentially impacted by on-site, small scale, and large scale waste water treatment facilities. Sites 1, 3, and 4 were impacted by OSSFs, site 2 by a small scale WWTP, site 5 by a large scale WWTP, and site 6 and 7 represented downstream surface water from the Fyris River and the Lake Ekoln (details about the sampling locations are shown in Table 1. For the sampling, POCIS were placed in a stainless steel basket and deployed at ~1 m under the water’s surface for two weeks. After sampling, the HLB bulk sorbent was transferred into an empty polypropylene cartridges (6 mL) containing two polyethylene (PE) frits (Supelco, St Paul, MN, USA) and then eluted using methanol (8 mL). The methanol extract was concentrated to 0.5 mL and the solvent changed to ethanol.

**Table 1:** Sample site ID, locations and impact by waste water facilities with population equivalent (PE).

Site	Location	Latitude	Longitude	Impact of existing sources for MPs
1	Upstream Björklinge village	60°01′59.35″N	17°27′52.96″E	OSSFs
2	Downstream Björklinge village and small scale WWTP	59°59′47.26″N	17°31′56.34″E	Small scale WWTP (3,700 PE)
3	Husby, tributary to Fyris River	59°52′6.20″N	17°36′12.03″E	OSSFs
4	Sävja River, tributary to Fyris River	59°49′55.12″N	17°39′38.18″E	OSSFs
5	Downstream Uppsala City and large scale WWTP	59°49′53.63″N	17°41′25.34″E	WWTP (172,000 PE)
6	Downstream the junction of Fyris River and Sävja River	59°48′33.76″N	17°40′7.80″E	WWTP (172,000 PE)
7	Lake Ekoln	59°45′26.45″N	17°38′15.86″E	Fyris River

Passive sampler extracts were diluted by a factor 100 in the cell culture medium and cells in all assays were thus exposed to a 100 times diluted passive sampler extract, defined as 1% of extract. The sample from site 3 in June 2015 was tested at 0.5% extract due to cytotoxicity at 1%.

From the Swedish Meteorological and Hydrological Institute the following data on precipitation in Uppsala were obtained: 35, 35, 44 and 73 mm for November 2014, March, June, and September 2015, respectively, and on average water flow rates in Fyris River during the same periods: 8.9, 30.8, 7.1 and 2.9 m/s, respectively.

### Cell lines and culturing conditions

All experiments were conducted in already established and commercially available cell lines and no animal experiments were conducted in this study.

Human hepatocellular carcinoma cell line HepG2 was cultured in Dulbecco’s modified Eagle medium (DMEM) (with 4.5 g/L glucose) (Lonza) supplemented with 2 mM L-glutamine (Lonza), 100 U/mL penicillin, 100 µg/mL streptomycin, 0.25 µg/mL amphotericin B (all from Gibco, Thermo Fisher Scientific), and 10% fetal bovine serum (FBS) (Gibco, Thermo Fisher Scientific). HepG2 cells were used for transient transfections to study AhR activities.

HepG2 cells stably transfected with an Nrf2 sensitive luciferase plasmid (Nrf2-HepG2) were purchased from Signosis (Santa Clara, CA, USA) and cultured as described above, with the addition of 100 µg/mL of Hygromycin B (InvivoGen).

HepG2 cells stably transfected with an NFκB sensitive luciferase plasmid (NFκB-HepG2) were purchased from Signosis (Santa Clara, CA, USA). Cells were cultured using the same cell culture media as described above with the addition of 100 µg/mL Hygromycin B (InvivoGen), but without addition of L-glutamine.

Human breast cancer cell line MCF-7 stably transfected with an estrogen receptor sensitive luciferase plasmid (VM7Luc4E2 cells)^24^ were routinely cultured in RPMI 1640 (Gibco) supplemented with 8% fetal bovine serum (FBS) (Gibco), 100 U/mL penicillin, 100 µg/mL streptomycin (Lonza), and 0.55 mg/mL Gentamicin (Lonza). For experiments, cells were cultured in DMEM (with 4.5 g/L glucose) (Lonza) supplemented with 4.5% dextran-charcoal treated fetal bovine serum (Thermo Scientific), 4 mM L-glutamine (Lonza), 100 U/mL penicillin, 100 µg/mL streptomycin (Lonza), and 0.38 mg/mL Gentamicin (Lonza).

Chinese hamster ovary cell line (CHO) stably transfected with an androgen receptor sensitive luciferase plasmid and an expression vector for the human androgen receptor (AR-Ecoscreen cells)^25,26^ were obtained from the Japanese Collection of Research Bioresources Cell Bank and routinely cultured in DMEM:F12 (Sigma) medium supplemented with 5% FBS (Gibco), 100 U/mL penicillin, 100 µg/mL streptomycin (Lonza), 2 mM L-glutamine (Lonza,), 100 µg/mL Hygromycin (InvivoGen), and 200 µg/mL Zeocin (Invitrogen). For experiments, cells were cultured in DMEM:F12 (Sigma-Aldrich) medium supplemented with 5% dextran-charcoal treated fetal bovine serum (Thermo Scientific), 2 mM L-glutamine (Lonza), 100 U/mL penicillin and 100 µg/mL streptomycin (Lonza).

All cells were cultured in a humidified environment at 37 °C and 5% CO_2_.

### Cell viability

To assay the cell viability in HepG2 cells and AR-Ecoscreen cells, the capacity of the cells to reduce a tetrazolium compound (MTS) to the colored formazan was analyzed. HepG2 cells were seeded in a density of 15,000 cells per well in a 96 well plate. Medium was exchanged 48 h after seeding and after an additional 24 h the cells were exposed to the water samples in a concentration of 1% of the passive sampler eluate.

AR-Ecoscreen cells were seeded in a density of 9,000 cells per well in a 96 well plate. After 24 h, the cells were exposed to the water samples in a concentration of 1% of the passive sampler eluate.

24 h after initiation of exposure, the Celltiter 96 AQueous One Solution Reagent (Promega) was added and the cell viability was then assayed in accordance with the manufacturer’s instructions using a Wallac Victor2 1420 microplate reader (PerkinElmer) and relative effects on cell viability was calculated in relation to the vehicle control.

For VM7Luc4E2 cells, the cell viability was analyzed using the CellTiter-Glo Luminescent Cell Viability Assay (Promega). For this assay, cells were seeded in a density of 40,000 cells per well in a 96 well plate and incubated for 24 h and successively exposed to water samples in a concentration of 1% of the passive sampler eluate for 24 hours. After the exposure, the cell viability assay substrate was added and the luminescence was measured in a Wallac Victor2 1420 microplate reader (PerkinElmer) and relative effects on cell viability was calculated in relation to the vehicle control.

### Reporter gene assays

The activity of AhR was studied using transiently transfected HepG2 cells. Cells were seeded in a density of 15,000 cells per well in a 96 well plate using the cell culture medium described above.The cells were incubated for 48 h before transfection with 30 ng/well of renilla plasmid and 90 ng/well of an AhR responsive luciferase plasmid (PGudLuc7.5)^27^. This plasmid was a kind gift from Professor Michael Denison, University of California at Davis. The DNA was delivered in 10 µL/well of Opti-MEM (1x) reduced serum medium (Gibco) with 0.3 µL/well Lipofectamine 2000 Reagent (Invitrogen by Life Technologies), as recommended by the manufacturer. Following the transfection, the cells were incubated for 24 h and then exposed to water samples or positive controls. Following 24 h of treatment, the reporter activity was measured using Dual-Luciferase Reporter 1000 Assay System (Promega) essentially according to the manufacturer’s protocol.

The activities of Nrf2, NFκB, ER, and AR were analyzed using cell lines stably transfected with luciferase plasmids sensitive to the respective receptor or transcription factor. Nrf2-HepG2, NFκB-HepG2, and AR-Ecoscreen were seeded in 96 well plates at a density of 17,000 cells, 10,000 cells or 9,000 cells per well, respectively, and cultured for 24 h. VM7Luc4E2 cells were seeded in 96 well plates at a density of 40,000 cells per well in estrogen free exposure media without gentamicin for 24 h. The cells were then exposed to water samples or positive controls. AR antagonistic effects of water samples were assessed by co-treating cells with 500 pM 5α-dihydrotestosterone. Following 24 h of treatment, the reporter activity was measured using Luciferase Reporter Assay System (Promega) according to the manufacturer’s protocol. The luminescence for both stably and transiently transfected cells was analyzed using an Infinite M1000 plate reader (Tecan) measuring luminescence for both renilla and firefly luciferase reaction over a 5 s period. The luciferase activity was expressed as fold change compared to the control treated group. To account for differences in transfection efficiency for transiently transfected cells, the reporter activity was standardized to the activity of the renilla plasmid for each well.

GR mediated cross talk may occur in the AR-Ecoscreen cells^26^. To exclude the possibility that induction of AR activity was rather due to glucocorticoid cross talk, positive samples were co-treated with the AR antagonist hydroxyflutamide in a concentration of 1 µM.

For each assay, a positive control was included as a standard curve; 2, 3, 7, 8-tetrachlorobenzo-p-dioxin (TCDD) (SUPELCO, Sigma-Aldrich) for AhR, tert-butylhydroquinone (tBHQ) (Sigma- Aldrich) for Nrf2, TNFα (Gibco) for NFκB, and 17β-estradiol (E2) (Sigma-Aldrich) for ER. 5α-dihydrotestosterone (DHT) (Sigma-Aldrich) was used as standard in AR agonistic assay and hydroxyflutamide (Sigma-Aldrich) was used as a standard in the AR antagonism assay.

### Data evaluation

The data evaluation was performed as previously described^28^. In short, treatment groups were normalized to vehicle control. The vehicle control was set to 1. Classification of samples as positive in the bioassays were based on cut-off values calculated from the limit of detection (LOD). For ER, AR, and AhR agonistic activities and Nrf2 induction, the LOD was defined as 1 plus 3 times the standard deviation (SD) of the vehicle control (Table 2). The LOD for the AR antagonist activity was calculated as 1 minus 3 times the SD. The Cut-off values for classification as positive sample for ER and AR activities were based on the OECD guidelines^24,26^. For ER agonism, the cut-off value was defined as 20% of E2 maximum response. For AR agonism, the cut-off value was defined as 10% of DHT maximum response. For AR antagonist activity, 30% reduction of the 500 pM DHT control was used as the definition of positive sample. For cytotoxicity, <0.8 of cell viability compared to a vehicle control set at 1, was used as a definition of cytotoxic effects.

**Table 2:** Derived curve fit parameters for positive controls (EC50, slope), reporter gene assay limit of detection, OECD criteria for classification of positives, and decided cut-off values in this study.

Assay	Positive control	EC50	Slope^b^	Max^a^	LOD^c^	OECD^d^	Cut-off
		mol/L		Fold change			
AhR	TCDD	1.1*10^−9^	1.6	31.2	1.6	—	2
NFkB	TNFα	—	—	—	1.4	—	1.5
Nrf2	tBHQ	1.9*10^−5^	1.8	23.9	1.7	—	2
AR agonism	DHT	3.6*10^−10^	1.9	6.3	1.3	1.5	1.5
AR antagonism	OHF	2.1*10^−8^	−0.3	−0.002	0.7	0.7	0.7
ER	E2	1.6*10^−10^	1.2	4.2	1.6	1.6	1.6

^a^Maximum estimated response of positive control.

^b^Hill slope.

^c^LOD = limit of detection, determined as described in section 2.6 and based on vehicle controls from experiments presented in Figs 1 and 2 (n = 12–16).

^d^Based on estimated maximum response of standards according to the OECD guidelines (OECD, 2016, 2012) as described in section 2.6.

The response of positive control compounds were modelled by a four parameter sigmoidal curve fit, except for TNFα, which was not fitted. For the curve fit, top/bottom was constrained at 1. The estimated EC50, Hill slope, and maximum response based on the curve fit for TCDD and E2 were used to convert water sample responses into equivalent concentrations of TCDD and E2 by inserting water sample responses in the following equation,

$${log}\,(concentration)=\,{log}(E{C}_{50})-\frac{log(\frac{top-response}{response-bottom})}{hillslope}$$

TCDD and E2 equivalent concentrations are only calculated for samples above the determined cut-off value.

### Chemical analysis

A DIONEX UltiMate 3000 ultra-performance liquid chromatography (UPLC) system (Thermo Scientific, Waltham, MA, USA) coupled to a triple quadrupole mass spectrometer (MS/MS) (TSQ QUANTIVA, Thermo SCIENTIFIC, Waltham, MA, USA) was used for the instrumental analysis, as previously described^29^. Separation by chromatography was performed using an Acquity BEH 1.7 *µ*m C18 column (Waters Corporation, UK). A guard column with the same packaging material preceded the separation column. The Thermo Xcalibur the MS system with TSQ Quantiva Tune Application softwares were used to operate the UPLC system. Thermo TraceFinder General LC software was used for data processing. Simultaneous positive and negative electrospray ionisation mode was used for acquisition. The organic phase consisted of acetonitrile while the aqueous phase was 5 mM ammonium acetate buffer. Flow rate was 0.5 mL min^−1^, with a column temperature set to 40 °C. The total run time was 15 min and the injection volume 10 µL.

Quantification, based on peak areas, was performed by the internal standard calibration approach. For each compound, its corresponding deuterated compound was used for quantification, except for those substances whose labelled analogue was not available. In this case, the most similar deuterated compound, in terms of chemical structure and chromatographic retention time, was used as IS. Table SI1 in the Supplementary Information shows the IS used for the quantification of each compound. The Commission Decision 2002/657/EC was used for identification and confirmation criteria’s for the analysis of target compounds.

## Results

### Cell viability

The water samples did not lead to compromised cell viability above the cut-off value at the treatment concentration of 1% of the passive sampler eluate, except the sample collected at site 3 in June 2015, which led to toxicity just below the cut-off level at 1%, but not at 0.5% of passive sampler eluate in AR-Ecoscreen cells (Fig. SI1, Supplementary Information). All activities presented below are examined at non-cytotoxic concentrations.

### Standards and cut-off values

**Figure 1:**
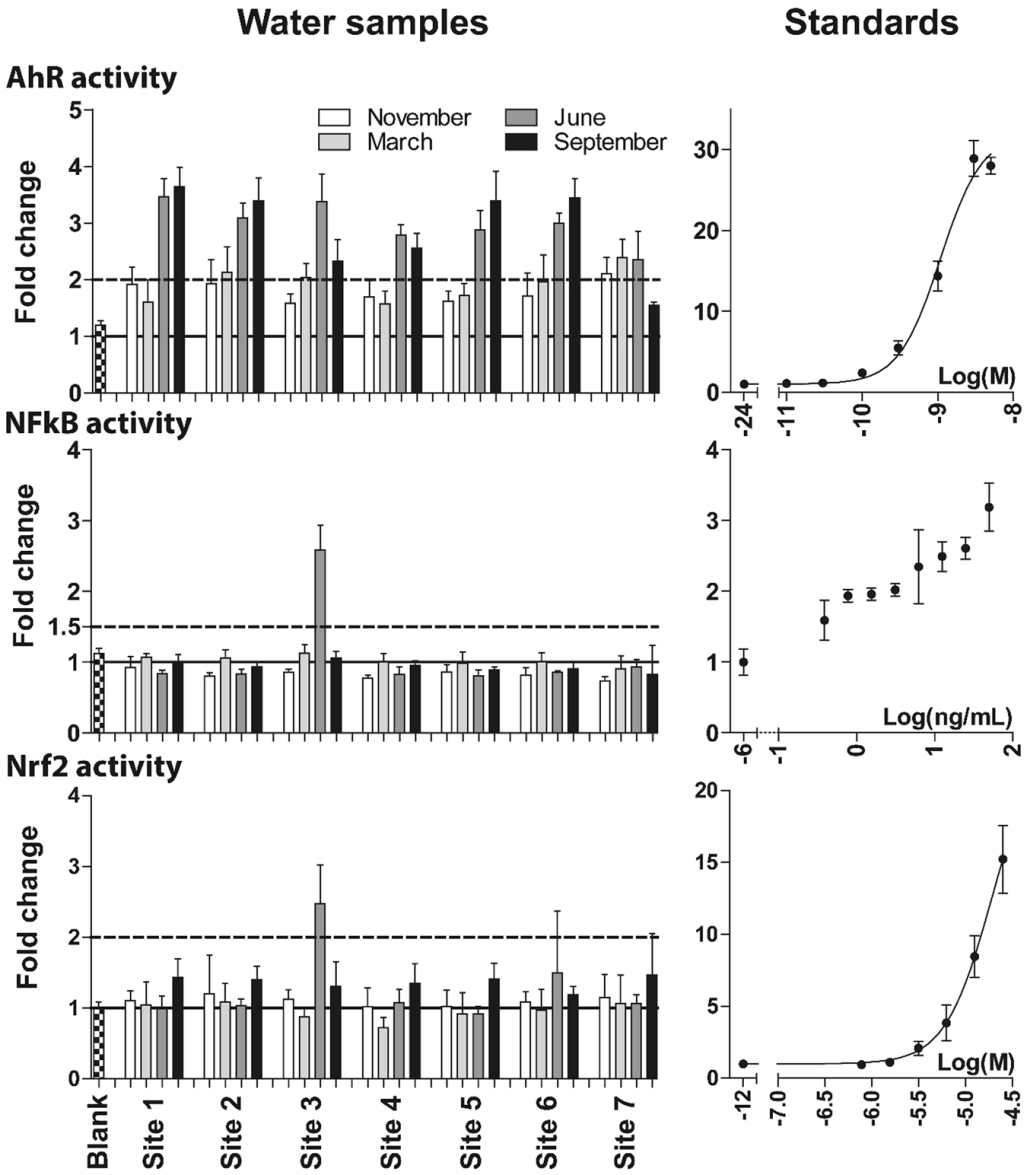
Activity induced by passive water samples (POCIS) deployed at 7 sampling sites over four seasons for the aryl hydrocarbon receptor (AhR) (n = 4), nuclear factor kappa-light-chain-enhancer of activated B cells (NFkB) pathway (n = 4), and nuclear factor erythroid 2-related factor 2 (Nrf2) pathway (n = 4) reporter gene assays and respective standard curves for TCDD (n = 3), TNFα (n = 3), and tBHQ (n = 3). TCDD and tBHQ were fitted to a four parameter sigmoidal curve fit with the bottom constrained to 1. Activities were normalized to vehicle control (n = 8), set at 1 (solid line). Dotted lines indicate cut-off values. Bars represent mean ± SD. Seasonal samples are indicated in white (November), light grey (March), dark grey (June), and black (September).

**Figure 2:**
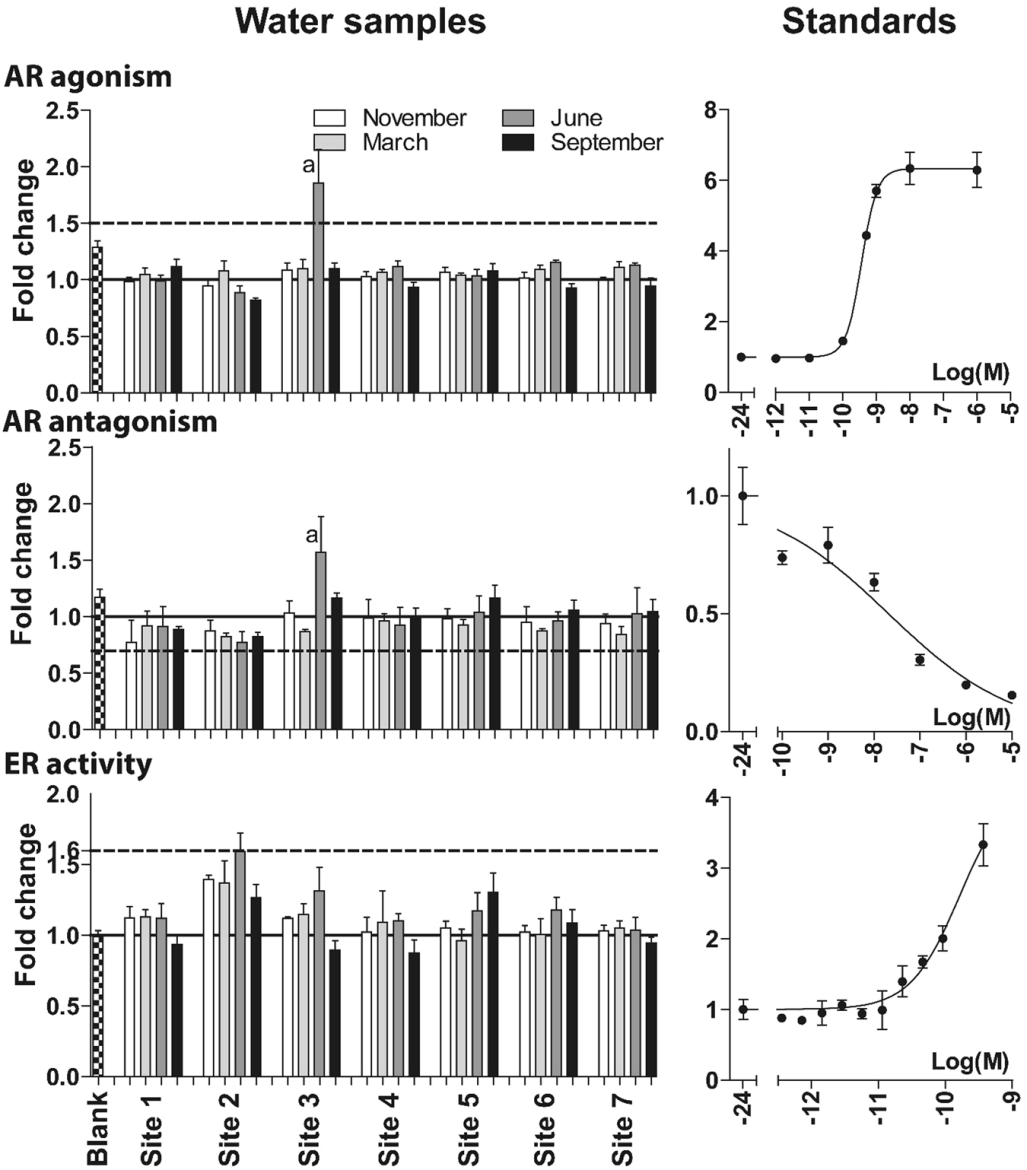
Activity induced by passive water samples (POCIS) deployed at 7 sampling sites over four seasons for the androgen agonist receptor (AR agonist) (n = 4), androgen antagonist receptor (AR antagonism) (n = 4), and estrogen receptor (ER) (n = 3) reporter gene assays and respective standard curves for DHT (n = 3), OHF (n = 3), and E2 (n = 2). Standards were fitted to a four parameter sigmoidal curve fit with the top/bottom fixed at 1. Activities were normalized to vehicle control (n = 6–8), set at 1 (solid line). Dotted lines indicate cut-off values. Bars represent mean ± SD. Seasonal samples are indicated in white (November), light grey (March), dark grey (June), and black (September). All samples were tested at 1% of original material, except the June sample from site 3, which was non-cytotoxic at 0.5% in AR-EcoScreen cells. a = sample tested at 0.5% of eluate due to compromised cell viability at 1%.

Standard curves for known inducers/inhibitor for the respective reporter gene assays are shown in Figs 1 and 2. Data for determination of cut-off levels for classification as a positive result in the bioassays are presented in Table 2. For bioassays lacking OECD guideline, the cut-off was defined based on the LOD. For assays with a LOD <1.5, the cut-off value was defined as 1.5. For assays with a LOD in the range 1.5–2, the cut-off values was defined as 2. For AR and ER agonism, the cut-off values were set to 1.5 and 1.6, respectively. For AR antagonism, the cut-off value was set to 0.7. These cut-off values were above or equal to the LODs and in accordance with the guidelines defined by OECD.

The blank sample, prepared with ultrapure water and included in all assays, gave responses comparable to the vehicle control (Figs 1 and 2).

### Bioactivity of passive water samples

Of the examined endpoints, most positive samples were observed in the AhR reporter gene assay from all the sampled sites, whereas only a few passive water samples induced activities in the remaining assays (Figs 1 and 2). For sampling sites 1–6, the June and September samples were consistently above the cut-off value for the AhR reporter assay and exhibited higher activities than samples from November and March. Sampling season seemed to have a higher impact on AhR activity than the sampling site. The pattern for site 7, where the river system has merged into Lake Ekoln and been significantly diluted, was different. November, March and June samples exhibited activities of 2–2.5 fold induction, and the September sample activity were below the cut-off value, approximately 1.5 fold induction.

In the remaining reporter gene assays, mostly no activity was observed. However, the June sample from site 3 (impacted by OSSFs) exhibited activities above the cut-off in all assays, except the ER reporter gene assay (Figs 1 and 2). This passive water sample activated the AR in both the antagonist and agonist mode of the AR reporter gene assay with similar efficacy of ~1.7 fold induction. Samples activating AR in both agonist and antagonist mode might, according to the OECD guideline^26^, actually be glucocorticoid receptor (GR) agonists. To test this hypothesis, we exposed AR-Ecoscreen cells to this sample together with the known AR antagonist hydroxyflutamide. If the sample is activating AR, the activity should be decreased when adding an AR antagonist. We observed no difference in reporter activity when comparing the effect of the water sample alone (fold induction 2.1 ± 0.06) versus the water sample in co-treatment with hydroxyflutamide (fold induction 2.2 ± 0.08). Thus, the observed reporter activity by the June sample from site 3 is not AR mediated but possibly due to GR activation. Additionally, the June sample from site 2 showed activity at the 1.6 fold-induction cut-off in the ER reporter gene assay (Fig. 2).

Passive water samples responses at or above the cut-off value in the AhR and ER reporter gene assay, were converted into TCDD and E2 equivalents based on the sigmoidal curve fits (Table 3 for TCDD equivalents). The observed effects were equivalent to an amount of 4.2–7.8 ng TCDD per sampler and 1.3 ng E2 per sampler for the June sample from site 2.

**Table 3:** TCDD equivalents for passive water samples.

Site ID	Season	TCDD/sampler (ng)
Blank		—
1	NOV	—
1	MAR	—
1	JUN	7.4
1	SEP	7.8
2	NOV	—
2	MAR	4.4
2	JUN	6.6
2	SEP	7.3
3	NOV	—
3	MAR	4.2
3	JUN	7.2
3	SEP	4.9
4	NOV	—
4	MAR	—
4	JUN	6
4	SEP	5.5
5	NOV	—
5	MAR	—
5	JUN	6.2
5	SEP	7.3
6	NOV	—
6	MAR	—
6	JUN	6.4
6	SEP	7.4
7	NOV	4.4
7	MAR	5.1
7	JUN	5
7	SEP	—

^1^TCDD equivalents are calculated based on the estimated EC50, Hill slope, and maximum response from the four parameter sigmoidal curve fit for TCDD. (—) response not above cut-off value of 2-fold induction and thus no TCDD equivalents were calculated.

### Occurrence of micropollutants

**Figure 3:**
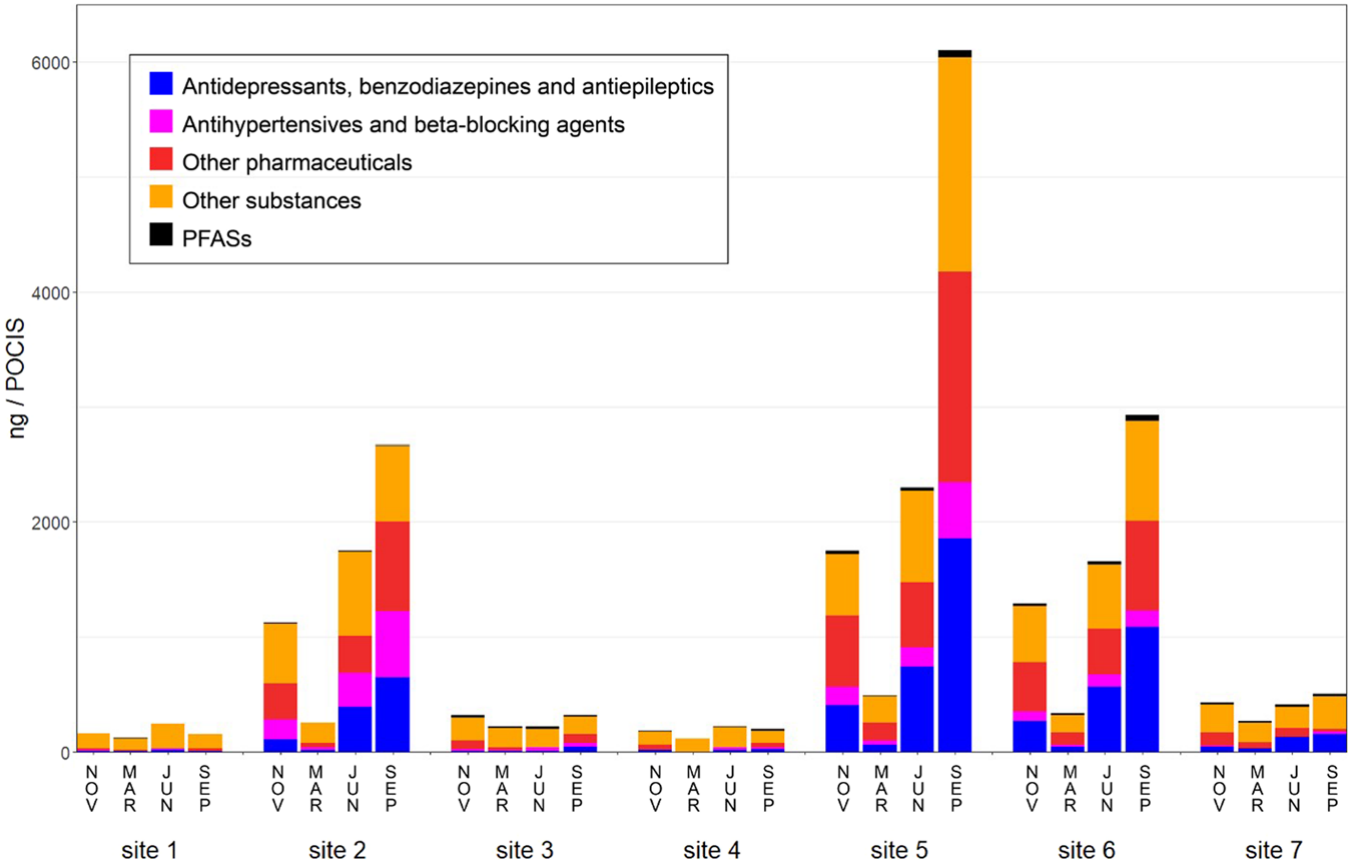
Concentrations of micropollutants derived from the passive samplers (ng/POCIS sampler) at seven sampling sites sampled in November (2014), March (2015), June (2015), and September (2015). Micropollutants were subdivided into five categories: I) antidepressants, benzodiazepines, and antiepileptics, II) antihypertensives and beta-blocking agents, III) other pharmaceuticals, IV) other substances, and V) perfluoroalkyl substances (PFASs).

The occurrence of the MPs was evaluated, and 58 of the 80 targeted substances were detected in at least one of the passive water samples. The analytes were divided into five different categories, where the first three categories include only pharmaceuticals: (I) antihypertensives and beta-blocking agents; (II) antidepressants, benzodiazepines and antiepileptics; (III) other pharmaceuticals, including antibiotics, analgesics, NSAIDs and antifungals, among others; (IV) PFASs and (V) “other substances” including illicit drugs, artificial sweeteners, pesticide, stimulants and personal care products. Residues of compounds belonging to the five described categories were detected in all the analysed passive samplers. The total amount of the different categories of MPs in each passive water sample is presented in Fig. 3. The specific amount of each compound in each passive water sampler is presented in Table SI2 in the Supplementary Information. The occurrence of MPs showed a clear spatial trend, with the highest number and total amount of MPs detected in the samples collected downstream of the two WWTPs included in this study (sites 2, 5, and 6), where up to 47 MPs were detected in one single sample. Total concentrations of up to 6100 ng/passive water sampler were detected (site 5, September). The Fyris River catchment drains into Lake Ekoln and is thereby diluted, explaining the lower MP levels at site 7 compared to site 6. Sample sites affected mainly by OSSFs showed lower levels ranging from 108–322 ng/passive water sampler (sites 1, 3, 4) compared to sites affected by a small scale WWTP (site 2) (228–2666 ng/sampler) and a large scale WWTP (sites 5 and 6) (335–6100 ng/sampler). For the sampling sites downstream of the WWTPs (sites 2, 5, and 6), we observed a clear seasonal pattern, with the total concentrations of MPs decreasing by a factor of ~4–5 from November to March and then increasing by a factor of ~5–13 in June and September. However, those seasonal patterns were not observed for the sites mainly impacted by OSSFs (sites 1, 3, 4).

## Discussion

In this study, we performed an integrated toxicological and chemical characterization of passive water samples from seven sampling sites in a river system, all differentially impacted by OSSFs and/or WWTPs. The methodology of passive sampling allows an integrative sampling over extended time, which is of great value for environmental sampling where the contaminant concentration might fluctuate^30^, however, in the future a better understanding of the uptake kinetic of passive samplers is needed to improve the comparison with concentrations and effect concentrations obtained by grab sampling methods. We observed bioactivity in all bioassays, in at least one of the samples, whereas the blank sample did not show bioactivity in any of our assays, showing that there are no false positive results due to contamination from the passive samplers or the sample preparation.

Activation of AhR was observed in samples from all sites, showing that AhR is a responsive endpoint for toxicity screening of environmental water samples. There was a tendency of a lower response in samples collected in Lake Ekoln (site 7), which can be explained by the fact that the riverine water is diluted in the lake water. All the sampling sites in the river system (sites 1–6) showed a similar profile regarding AhR activity, while there was a large variation in total amount of analyzed MP:s ranging from 108–322 ng/sampler for sites impacted by OSSF compared to 335–6100 ng/sampler for sites impacted by WWTPs. This indicates that the AhR activity is not caused by the total mixture of measured MPs from OSSFs or WWTPs. Possible inducers of AhR activity in the water samples may be other environmental pollutants or naturally occurring compounds^31,32^.

We observed a seasonal pattern for the AhR activity in all samples collected within the river system, with the highest activities detected in samples collected in June and September. These two months had the highest amount of precipitation. The higher AhR activity during rainy periods may be due to AhR inducing compounds, such as polycyclic aromatic hydrocarbons (PAHs) from difference source pathways such as surface run-off, atmospheric deposition and storm water.

The AhR activities observed are equivalent to a TCDD amount of 4.2–7.8 ng per sampler. Jálová *et al*.^33^ reported similar results from analysis of passive water samples collected with POCIS samplers in the Svratka River system (Czech Republic), where the samples positive for AhR activity were found to have an activity corresponding to up to 2 ng TCDD per sampler, suggesting that the water samples in the present study exhibit bioactivity in the same range as that previously reported.

Other studies have used passive sampling of water coupled to *in vitro* bioassays for analysis of potential toxicity. The observed toxicity profile of the samples varies greatly between the studies, which could be expected since they have been performed in different rivers systems and countries, where MPs may vary greatly in concentration. Furthermore, differences between studies might also be due to differences in deployment time and/or type of passive sampler. Tapie *et al*.^34^ observed ER activity, but not AhR activity, in the extract from a POCIS passive sampler that had been deployed in the Baïse River in south-west France. In a study evaluating different treatment methods in a WWTP, Escher *et al*.^35^ reported that extracts from syrene divinylbenzene-reverse phase sulfonated Empore extraction disk passive samplers generally did not show bioactivity above the limit of quantification for a number of bioassays. For the effects they did observe, only 1% or less could be explained by the chemical characterization performed for the same samples. A study performed in the River Ythan catchment in north-east Scotland observed increased EROD activity, as a measurement of AhR activity, in all five samples collected with silicone rubber passive samplers^36^. In our study, we observed a decrease in the AhR activity when the rivers system flows into a lake and the river water is diluted in the lake water. The same has been observed by Li *et al*.^37^ in a study where polydimethylsiloxane (PDMS) passive samplers were used to study the bioactivity in Brisbane River, Oxley Creek, and Port of Brisbane, Australia. In similarity with our results, Jálová *et al*.^33^ did not observe ER or AR activity in extracts from POCIS passive samplers that had been deployed in a river system in the Czech Republic. However, they observed AhR activity in extracts from passive samplers that had been deployed downstream, but not upstream, of a WWTP.

The occurrence of MPs in the samples showed a spatial pattern, with the highest amounts of MPs detected downstream of the large scale WWTPs, while the detected amounts at OSSF sites were considerably lower. We also observed a seasonal pattern in the samples with a high impact from WWTPs, with the highest amount of MPs in September and lowest amounts in March, which may be explained by a low river flow and less dilution of the effluents from the WWTPs in September and a high river flow and a higher dilution in March.

A comparison between the occurrence of MPs and the observed AhR activity shows that the AhR activity is high also in samples with low amounts of MPs. The lack of correlation clearly indicates that the observed AhR activity cannot be explained by the measured MPs. It has been repeatedly reported by other groups that chemical analysis of a targeted group of MPs can only explain a relatively limited portion of the observed bioactivity^11,13,35,38,39^, lending support to the use of bioactivity measures to obtain knowledge on the total toxic potential of a water sample and not only rely on chemical analysis. Combined fractionation and non-target screening could be a suitable strategy to identify the chemicals causing this bioactivity.

Interestingly, we generally did not observe estrogenic activity in the water samples, not even in the samples collected downstream of a large-scale WWTP serving approximately 172,000 population equivalents (PE). For other geographical locations, estrogenicity has repeatedly been reported for WWTP effluent water and/or surface water downstream of WWTPs^19,37,40–43^.

One single water sample, collected at site 3 in June, showed bioactivity in multiple bioassays; AhR, NFĸB, Nrf2 and AR agonist activity. Site 3 is impacted by OSSFs, but not by any large scale waste water treatment facilities or large-scale industries. The analyzed MPs cannot explain the observed bioactivity. All detected MPs in this sample were present in low amounts, and detected in higher amounts in other samples that did not show bioactivity in these assays. The OECD guideline 458^26^ specifies that a sample that shows AR activity in both the AR agonist and AR antagonist mode of the assay, might be an glucocorticoid receptor (GR) agonist, acting via GR cross talk. We tested this hypothesis by co-treatment with the known AR antagonist hydroxyflutamide, in accordance with the OECD guideline, and found that the observed effect is non-AR mediated, but possibly through activation of GR. At present, we are unable to explain which compound(s) that might cause the observed bioactivity of this water sample.

In conclusion, we have studied both the toxicity and the occurrence of MPs in passive water samples differentially impacted by OSSFs and WWTPs. The occurrence of MPs showed a clear spatial pattern, with the highest amounts downstream of WWTPs while the amounts of MPs were considerably lower at sites impacted by OSSFs. However, the AhR activity did not differ between the river sampling sites.

## Electronic supplementary material

Supplementary information

## Data Availability

The datasets generated during the current study are available from the corresponding author upon request.
