# Supplementary material for: Effect-based assessment of recipient waters impacted by on-site, small scale, and large scale waste water treatment facilities – combining passive sampling with in vitro bioassays and chemical analysis
Source: Sci Rep. 2018 Nov 21;8:17200. doi: 10.1038/s41598-018-35533-x (PMC6249289; doi:10.1038/s41598-018-35533-x)
Supplement: Supplementary file 1 — Supplementary information [file 41598_2018_35533_MOESM1_ESM.pdf]

Supplementary information

Effect-based assessment of recipient waters impacted by on-site, small scale, and large scale waste water treatment facilities – combining passive sampling with *in vitro* bioassays and chemical analysis

Anna Kjerstine Rosenmai[a], Johan Lundqvist[a], Pablo Gago-Ferrero[b], Geeta Mandava[a], Lutz Ahrens[b], Karin Wiberg[b], and Agneta Oskarsson[a]

[a]Department of Biomedical Sciences and Veterinary Public Health, Swedish University of Agricultural Sciences, Box 7028, SE-750 07 Uppsala, Sweden
[b]Department of Aquatic Sciences and Assessment, Swedish University of Agricultural Sciences, Box 7050, SE-750 07 Uppsala, Sweden

Figure SI1: Cell viability as determined by the MTS assay in VM7Luc4E2, HepG2, and AR-EcoScreen cells.

Table SI1: Names, CAS numbers and internal standards for the selected micropollutants.

Table SI2: Specific amount of each compound in each passive water sampler.

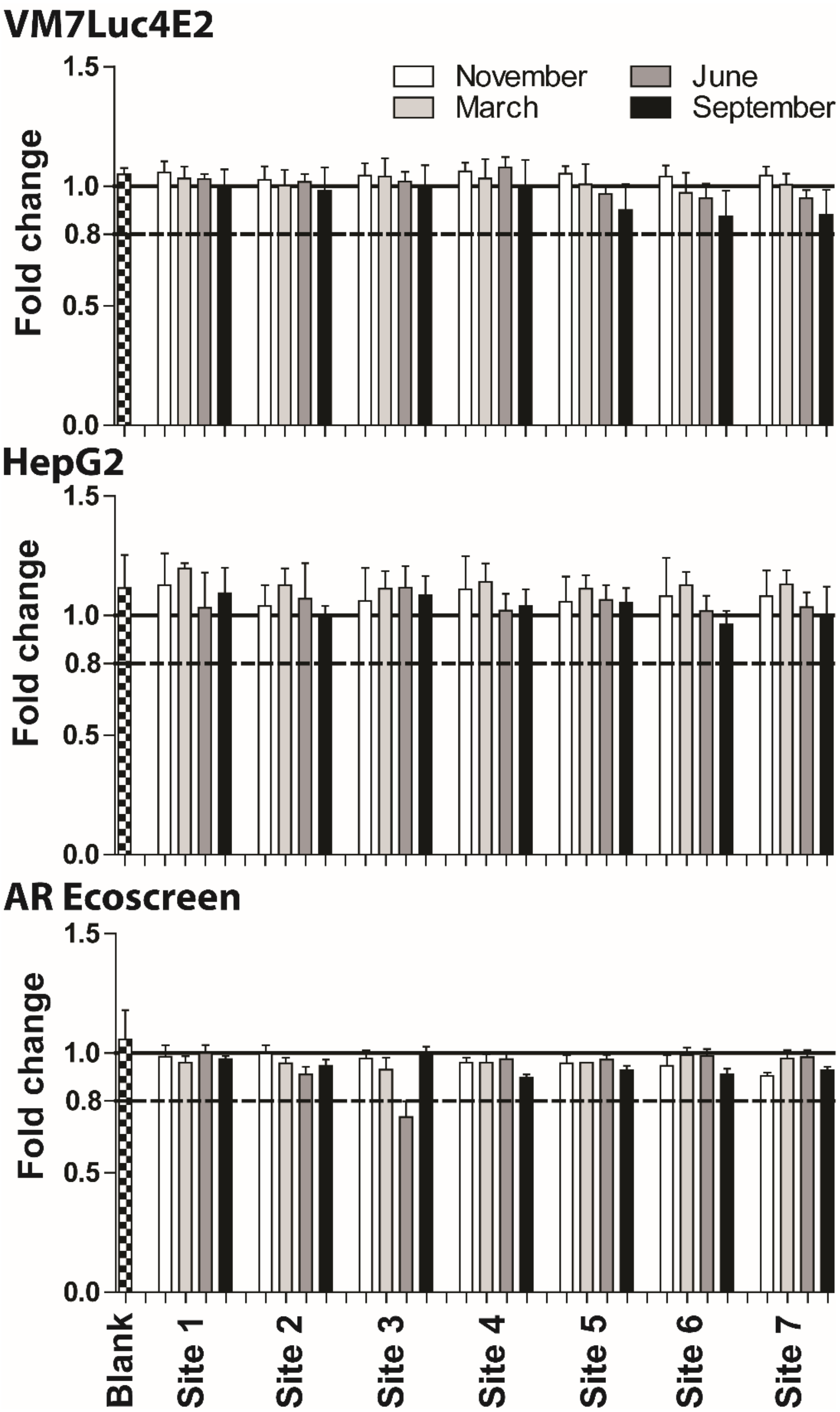

Figure S1: Cell viability as determined by the MTS assay in VM7Luc4E2, HepG2, and AR-EcoScreen cells. Water sample data (n=4) was normalized to the plate vehicle control (n=8) set to 1 (solid line). A cut-off of 0.8 fold-change was set as the limit for cytotoxicity (dotted line). None of the water samples caused cytotoxicity, except the June sample from Site 3. This sample was successively tested in dilutions and was determined non cytotoxic at 0.5% of original material. Only non-cytotoxic concentrations are presented in Figure 1 and 2.

Table SI1: Names, CAS numbers and internal standards for the selected micropollutants.

Category	Class	Compound	CAS number	Corresponding Internal Standard (IS)
**Pharmaceuticals (Antibiotics)**	III	Amoxicillin	26787-78-0	*Cis-Sertraline-D3*
	III	Azithromycin	83905-01-5	*Erythromycin-D3-13C*
	III	Ciprofloxacin	85721-33-1	*Ciprofloxacin-D8*
	III	Clarithromycin	81103-11-9	*Erythromycin-D3-13C*
	III	Erythromycin	114-07-8	*Erythromycin-D3-13C*
	III	Metronidazole	443-48-1	*Metronidazole-D4*
	III	Norfloxacin	70458-96-7	*Ofloxacin-D3*
	III	Ofloxacin	82419-36-1	*Ofloxacin-D3*
	III	Roxithromycin	80214-83-1	*Erythromycin-D3-13C*
	III	Sulfamethoxazole	723-46-6	*Sulfamethoxazole-D4*
	III	Trimethoprim	738-70-5	*Trimethoprim-D9*
	III	Tetracycline	60-54-8	*Erythromycin-D3-13C*
**Pharmaceuticals (analgesics)**	III	Acetaminophen (Paracetamol)	103-90-2	*Acetaminophen-D4*
	III	Tramadol	27203-92-5	*Tramadol-D3-13C*
	III	Morphine	57-27-2	*Codeine-D3*
**Pharmaceuticals (Anesthetics)**	III	Lidocaine	137-58-7	*Lidocaine-(diethyl)-D10*
**Pharmaceuticals (Antidepressants)**	II	Amitriptylline	50-48-7	*Carbamazepine-(carboxamide-13C,15N)*
	II	Citalopram	59729-33-9	*Oxazepam-D5*
	II	Desvenlafaxine	93413-62-8	*Venlafaxine-D6*
	II	Fluoxetine	54910-89-4	*Fluoxetine-D5*
	II	Sertraline	79617-96-3	*Cis-Sertraline-D3*
	II	Venlafaxine	93413-69-6	*Venlafaxine-D6*
	II	Mirtazapine	85650-52-8	*Fluoxetine-D5*
	II	Paroxetine	61869-08-7	*Fluoxetine-D5*
**Pharmaceuticals (Antiepileptics)**	II	Carbamazepine	298-46-5	*Carbamazepine-(carboxamide-13C,15N)*
	II	Lamotrigine	84057-84-2	*Lidocaine-(diethyl)-D10*
	II	Valproic acid	99-66-2	*Mefenamic Acid 13C6*
**Pharmaceuticals (Antidiabetics)**	III	Metformin	657-24-9	*Metronidazole-D4*
**Pharmaceuticals (Antihypertensives)**	I	Atenolol	29122-68-7	*Atenolol-D7*
	I	Diltiazem	42399-41-7	*Diltiazem-D4*
	I	Irbesartan	138402-11-6	*Irbesartan-D7*
	I	Losartan	114798-26-4	*Losartan-D4*
	I	Metoprolol	51384-51-1	*Atenolol-D7*
	I	Valsartan	137862-53-4	*Irbesartan-D7*

**Pharmaceuticals (Antilipidemic agents)**	III	Atorvastatin	134523-00-5	*Atorvastatin-D5*
**Pharmaceuticals (Antiulcers drugs)**	III	Omeprazole	73590-58-6	*Metronidazole-D4*
	III	Ranitidine	66357-35-5	*Ranitidine-D6*
**Pharmaceuticals (Antifungal)**	III	Climbazole	38083-17-9	*Metronidazole-D4*
	III	Fluconazole	86386-73-4	*Metronidazole-D4*
**Pharmaceuticals (Benzodiazepines)**	II	Diazepam	439-14-6	*Diazepam-D5*
	II	Oxazepam	604-75-2	*Oxazepam-D5*
**Pharmaceuticals (Beta blocking agents)**	I	Propranolol	525-66-6	*Atenolol-D7*
	I	Sotalol	3930-20-9	*Atenolol-D7*
**Pharmaceuticals (Diuretics)**	III	Furosemide	54-31-9	*Furosemide-D5*
	III	Hydrochlorothiazide	58-93-5	*Hydrochlorothiazide-D2-13C*
**Pharmaceuticals (Lipid lowering agent)**	III	Bezafibrate	41859-67-0	*Bezafibrate-D4*
**Pharmaceuticals (NSAIDs)**	III	Diclofenac	15307-86-5	*Diclofenac-13C6*
	III	Ibuprofen	15687-27-1	*Ibuprofen-D3*
	III	Meclofenamic acid	644-62-2	*Mefenamic Acid 13C6*
	III	Mefenamic Acid	61-68-7	*Mefenamic Acid 13C6*
	III	Niflumic acid	4394-00-7	*Mefenamic Acid 13C6*
**Artificial sweetener**	V	Sucralose	56038-13-2	*Sucralose-D6*
**Illicit drugs**	V	Cocaine (COC)	50-36-3	*Codeine-D3*
	V	Heroine	561-27-3	*Codeine-D3*
**Personal care products (Insect repellents)**	V	DEET (diethyltoluamide)	134-62-3	*DEET-D10*
**Personal care products (Parabens)**	V	Ethylparaben	120-47-8	*Propylparaben-D7*
	V	Methylparaben	99-76-3	*Propylparaben-D7*
	V	Propylparaben	94-13-3	*Propylparaben-D7*
**Personal care products (Sunscreens)**	V	Octocrylene	6197-30-4	*Octocrylene-D10*
	V	EHMC	5466-77-3	*Octocrylene-D10*
**Pesticides**	V	Isoproturon	34123-59-6	*Isoproturon-D3*
	V	Terbutryn	886-50-0	*Isoproturon-D3*
	V	BAM (Dichlorobenzamide)	2008-58-4	*DEET-D10*
**PFAS**	IV	perfluorobutane sulfonic acid (PFBS)	375-73-5	*PFHxS-18O2*
	IV	perfluorobutanoic acid (PFBA)	375-22-4	*PFBA-13C4*
	IV	perfluorodecanoic acid (PFDA)	335-76-2	*PFDA-13C2*
	IV	perfluorododecanoic acid (PFDoDA)	307-55-1	*PFDoDA-13C2*

	IV	perfluoroheptanoic acid (PFHpA)	375-85-9	*PFOA-13C4*
	IV	perfluorohexane sulfonic acid (PFHxS)	355-46-4	*PFHxS-18O2*
	IV	perfluorohexanoic acid (PFHxA)	307-24-4	*PFHxA-13C2*
	IV	perfluorononanoic acid (PFNA)	375-95-1	*PFNA-13C5*
	IV	perfluorooctane sulfonamide (FOSA)	754-91-6	*FOSA-M8*
	IV	perfluorooctane sulfonic acid (PFOS)	1763-23-1	*PFOS-13C4*
	IV	perfluorooctanoic acid (PFOA)	335-67-1	*PFOA-13C4*
	IV	perfluoropentanoic acid (PFPeA)	2706-90-3	*PFHxA-13C2*
	IV	perfluorotetradecanoic acid (PFTeDA)	376-06-7	*PFDoDA-13C2)*
	IV	perfluoroundecanoic acid (PFUnDA)	2058-94-8	*PFUnDA-13C2*
**Opiates, opioids and metabolites**	V	Codeine (COD)	76-57-4	*Codeine-D3*
	V	Oxycodone (OC)	76-42-7	*Codeine-D3*
**Stimulants**	V	Caffeine	58-08-02	*Caffeine-13C3*
	V	Nicotine	54-11-5	*Codeine-D3*

Table SI2: Specific amount of each compound in each passive water sampler.

POCIS (ng/Sampler)	LOD	LOQ	Field Blank	Sampling point 1				Sampling point 2				Sampling point 3				Sampling point 4				Sampling point 5				Sampling point 6				Sampling point 7			
				Nov-14	Mar-15	Jun-15	Sep-15	Nov-14	Mar-15	Jun-15	Sep-15	Nov-14	Mar-15	Jun-15	Sep-15	Nov-14	Mar-15	Jun-15	Sep-15	Nov-14	Mar-15	Jun-15	Sep-15	Nov-14	Mar-15	Jun-15	Sep-15	Nov-14	Mar-15	Jun-15	Sep-15
Acetaminophen	0.5	1.7		6.0	10.0	0.0	0.0	10.0	6.0	0.0	0.0	13.0	15.0	0.0	0.0	3.0	<LOQ	0.0	0.0	<LOQ	4.1	3.5	4.3	<LOQ	5.7	0.0	6.0	0.0	3.9	0.0	0.0
Amitryptilline	0.5	1.7		0.0	0.0	0.0	0.0	0.0	<LOQ	0.0	3.1	0.0	0.0	0.0	0.0	0.0	0.0	0.0	0.0	<LOQ	1.5	0.0	<LOQ	<LOQ	1.3	0.0	6.1	0.0	<LOQ	0.0	0.0
Amoxicillin	25	83.3		n.d.	n.d.	n.d.	n.d.	n.d.	n.d.	n.d.	n.d.	n.d.	n.d.	n.d.	n.d.	n.d.	n.d.	n.d.	n.d.	n.d.	n.d.	n.d.	n.d.	n.d.	n.d.	n.d.	n.d.	n.d.	n.d.	n.d.	n.d.
Atenolol	0.1	0.3		2.0	0.6	1.6	1.6	7.0	2.0	12.3	18.3	13.0	3.0	7.1	4.1	1.0	0.4	2.0	0.0	26.0	5.2	26.5	64.5	18.0	4.1	14.0	23.5	1.1	2.3	3.1	1.8
Atorvastatin	3.5	11.7		0.0	0.0	0.0	0.0	0.0	0.0	0.0	15.5	0.0	0.0	0.0	0.0	0.0	0.0	0.0	0.0	0.0	0.0	0.0	0.0	0.0	0.0	0.0	0.0	0.0	0.0	0.0	0.0
Azithromycin	0	0.0		n.d.	n.d.	n.d.	n.d.	n.d.	n.d.	n.d.	n.d.	n.d.	n.d.	n.d.	n.d.	n.d.	n.d.	n.d.	n.d.	n.d.	n.d.	n.d.	n.d.	n.d.	n.d.	n.d.	n.d.	n.d.	n.d.	n.d.	n.d.
BAM /Dichlorbenzamide	0	0.0		n.d.	n.d.	n.d.	n.d.	n.d.	n.d.	n.d.	n.d.	n.d.	n.d.	n.d.	n.d.	n.d.	n.d.	n.d.	n.d.	n.d.	n.d.	n.d.	n.d.	n.d.	n.d.	n.d.	n.d.	n.d.	n.d.	n.d.	n.d.
Bezafibrate	2.5	8.3		0.0	0.0	0.0	0.0	27.0	0.0	31.0	48.5	0.0	0.0	0.0	0.0	0.0	0.0	0.0	0.0	33.0	10.0	37.0	64.5	33.0	10.0	30.5	26.5	0.0	0.0	0.0	0.0
Caffeine	1	3.3		94.0	90.0	199.5	77.5	378.0	148.0	448.0	302.5	149.0	126.0	77.5	65.5	63.0	112.0	115.5	63.0	150.0	121.5	216.5	280.5	91.0	70.5	106.0	182.5	22.5	49.5	35.0	34.5
Carbamazepine	0.1	0.3		0.0	0.0	0.0	0.0	7.0	2.0	30.8	102.0	0.0	1.0	1.8	2.5	2.0	0.7	3.4	7.4	78.0	10.5	68.0	250.5	48.0	6.6	39.5	215.0	6.0	8.4	29.0	49.5
Ciprofloxacin	2.5	8.3		n.d.	n.d.	n.d.	n.d.	n.d.	n.d.	n.d.	n.d.	n.d.	n.d.	n.d.	n.d.	n.d.	n.d.	n.d.	n.d.	n.d.	n.d.	n.d.	n.d.	n.d.	n.d.	n.d.	n.d.	n.d.	n.d.	n.d.	n.d.
Citalopram	0.5	1.7		0.0	0.0	0.0	0.0	10.0	2.4	38.5	67.0	0.0	0.0	0.0	0.0	0.0	0.0	0.0	0.0	25.0	18.0	87.5	188.0	20.0	8.8	100.5	199.5	0.8	4.6	3.8	4.5
Clarithromycin	0.5	1.7		0.0	0.0	0.0	0.0	0.0	0.0	2.0	2.1	0.0	0.0	0.0	0.0	0.0	0.0	0.0	0.0	0.0	2.2	0.0	29.3	0.0	0.0	0.0	0.0	0.0	0.0	0.0	0.0
Climbazole	0.25	0.8		0.0	0.0	0.0	0.0	0.0	<LOQ	<LOQ	<LOQ	0.0	0.0	<LOQ	<LOQ	0.0	0.0	0.0	0.0	<LOQ	0.0	1.3	<LOQ	<LOQ	0.0	0.8	<LOQ	0.0	0.0	<LOQ	0.0
Cocaine	0.25	0.8		0.0	0.0	0.0	0.0	0.0	0.0	1.2	0.0	0.0	0.0	0.0	0.0	0.0	0.0	0.0	0.0	0.0	0.0	0.0	0.0	0.0	0.0	0.0	0.0	0.0	0.0	0.0	0.0
Codeine	0.25	0.8		0.8	0.0	1.5	1.8	14.0	2.4	70.0	69.0	1.0	1.1	1.0	0.8	<LOQ	0.0	<LOQ	<LOQ	18.0	4.2	74.0	152.5	18.0	3.8	22.4	52.5	1.0	1.6	2.6	<LOQ
DEET	0.1	0.3		0.0	0.0	<LOQ	7.6	2.0	0.0	5.6	16.7	0.0	0.0	2.5	7.6	4.0	0.0	0.9	1.5	16.0	0.5	14.0	7.3	14.0	0.0	6.3	5.6	1.7	0.0	0.4	4.3
Desvenlafaxine	1	3.3		<LOQ	<LOQ	4.9	0.0	25.5	4.0	147.5	205.5	<LOQ	<LOQ	0.0	0.0	4.0	0.0	6.8	10.0	58.0	3.3	112.0	150.5	43.0	2.0	96.0	93.0	10.9	<LOQ	46.0	22.5
Diazepam	0.5	1.7		n.d.	n.d.	n.d.	n.d.	n.d.	n.d.	n.d.	n.d.	n.d.	n.d.	n.d.	n.d.	n.d.	n.d.	n.d.	n.d.	n.d.	n.d.	n.d.	n.d.	n.d.	n.d.	n.d.	n.d.	n.d.	n.d.	n.d.	n.d.
Diclofenac	2.5	8.3		0.0	0.0	0.0	0.0	61.0	18.0	33.0	220.5	11.0	0.0	<LOQ	22.5	<LOQ	0.0	0.0	0.0	75.0	24.4	149.0	466.0	70.0	18.8	122.7	175.5	22.0	14.9	41.0	<LOQ
Diltiazem	0.25	0.8		n.d.	n.d.	n.d.	n.d.	n.d.	n.d.	n.d.	n.d.	n.d.	n.d.	n.d.	n.d.	n.d.	n.d.	n.d.	n.d.	n.d.	n.d.	n.d.	n.d.	n.d.	n.d.	n.d.	n.d.	n.d.	n.d.	n.d.	n.d.
EHMC	10	33.3		n.d.	n.d.	n.d.	n.d.	n.d.	n.d.	n.d.	n.d.	n.d.	n.d.	n.d.	n.d.	n.d.	n.d.	n.d.	n.d.	n.d.	n.d.	n.d.	n.d.	n.d.	n.d.	n.d.	n.d.	n.d.	n.d.	n.d.	n.d.
Erytthromycin	0.5	1.7		0.0	0.0	0.0	0.0	0.0	0.0	0.0	0.0	0.0	0.0	0.0	0.0	0.0	0.0	0.0	0.0	0.0	<LOQ	8.5	0.0	0.0	<LOQ	<LOQ	0.0	0.0	0.0	<LOQ	0.0
Ethylparaben	0.5	1.7		0.0	0.0	0.0	0.0	3.5	0.0	0.0	0.0	0.0	0.0	0.0	0.0	0.0	0.0	0.0	0.0	0.0	0.0	0.0	0.0	8.6	0.0	0.0	0.0	1.7	0.0	0.0	0.0
Fluconazole	1.5	5.0		0.0	0.0	0.0	0.0	<LOQ	0.0	<LOQ	0.0	0.0	0.0	0.0	0.0	0.0	0.0	0.0	0.0	<LOQ	0.0	0.0	0.0	<LOQ	0.0	0.0	0.0	0.0	0.0	0.0	0.0
Fluoxetine	0.25	0.8		n.d.	n.d.	n.d.	n.d.	n.d.	n.d.	n.d.	n.d.	n.d.	n.d.	n.d.	n.d.	n.d.	n.d.	n.d.	n.d.	n.d.	n.d.	n.d.	n.d.	n.d.	n.d.	n.d.	n.d.	n.d.	n.d.	n.d.	n.d.
Furosemide	4	13.3		0.0	0.0	0.0	0.0	99.0	<LOQ	104.0	252.5	27.0	0.0	0.0	44.5	26.0	0.0	0.0	38.5	155.0	45.5	130.0	435.5	121.0	25.0	85.5	156.0	47.0	0.0	0.0	0.0
Heroine	0.1	0.3		n.d.	n.d.	n.d.	n.d.	n.d.	n.d.	n.d.	n.d.	n.d.	n.d.	n.d.	n.d.	n.d.	n.d.	n.d.	n.d.	n.d.	n.d.	n.d.	n.d.	n.d.	n.d.	n.d.	n.d.	n.d.	n.d.	n.d.	n.d.
Hydrochlorothiazide	0.5	1.7		10.0	0.0	<LOQ	16.0	72.0	13.0	96.0	170.0	14.0	9.0	0.0	4.0	9.0	0.0	<LOQ	<LOQ	139.0	38.3	149.5	501.0	109.0	28.9	102.5	215.0	23.5	20.0	13.3	13.5
Ibesartan	5	16.7		0.0	0.0	0.0	0.0	24.0	0.0	40.5	56.5	0.0	0.0	0.0	0.0	0.0	0.0	0.0	0.0	22.0	0.0	36.0	152.5	0.0	0.0	34.5	57.0	0.0	0.0	0.0	0.0
Isoproturon	0	0.0		n.d.	n.d.	n.d.	n.d.	n.d.	n.d.	n.d.	n.d.	n.d.	n.d.	n.d.	n.d.	n.d.	n.d.	n.d.	n.d.	n.d.	n.d.	n.d.	n.d.	n.d.	n.d.	n.d.	n.d.	n.d.	n.d.	n.d.	n.d.
Lamotrigine	0.1	0.3		2.0	1.0	7.6	7.0	33.0	2.0	34.4	53.0	1.0	1.0	1.5	1.0	5.0	0.0	4.0	3.0	102.0	15.0	205.5	455.5	82.0	8.3	112.0	217.5	10.0	7.6	29.5	41.0
Lidocaine	0.1	0.3		1.0	0.2	0.0	0.0	13.0	2.0	6.0	8.3	8.0	1.0	4.0	5.5	2.0	0.0	0.0	<LOQ	61.0	16.6	53.0	210.5	42.0	12.8	37.5	106.0	7.7	10.1	10.5	7.8
Losartan	10	33.3		0.0	0.0	<LOQ	<LOQ	50.5	<LOQ	107.5	265.5	0.0	0.0	<LOQ	27.5	0.0	0.0	<LOQ	<LOQ	<LOQ	<LOQ	34.5	137.5	<LOQ	0.0	31.0	30.5	0.0	0.0	0.0	<LOQ
Meclofenamic acid	2.5	8.3		0.0	0.0	0.0	0.0	0.0	0.0	0.0	15.5	0.0	0.0	0.0	0.0	0.0	0.0	0.0	0.0	0.0	0.0	0.0	0.0	0.0	0.0	0.0	0.0	0.0	0.0	0.0	0.0
Mefenamic acid	1	3.3		0.0	0.0	0.0	0.0	0.0	0.0	0.0	0.0	0.0	0.0	0.0	0.0	0.0	0.0	0.0	0.0	0.0	0.0	3.1	12.5	0.0	0.0	0.0	0.0	0.0	0.0	0.0	0.0
Metformin	0.05	0.2		<LOQ	0.0	0.3	0.4	1.0	1.0	2.0	1.3	0.0	0.0	0.0	<LOQ	0.0	0.0	<LOQ	0.0	1.0	0.4	1.0	0.4	2.0	<LOQ	<LOQ	0.5	<LOQ	<LOQ	0.0	0.0
Methylparaben	0.5	1.7		9.4	<LOQ	0.0	0.0	22.0	11.0	12.5	18.5	4.3	4.0	12.0	12.0	7.1	0.0	0.0	0.0	4.6	<LOQ	6.0	7.5	10.4	0.0	0.0	0.0	2.4	0.0	0.0	0.0
Metoprolol	0.05	0.2		1.0	0.2	0.0	0.0	17.0	3.0	1.8	0.0	2.0	1.0	4.3	0.9	<LOQ	0.0	0.0	0.0	36.0	7.6	25.5	62.5	20.0	4.7	14.0	11.5	0.5	0.0	2.1	0.8
Metronidazole	2.5	8.3		0.0	0.0	0.0	0.0	0.0	0.0	0.0	0.0	0.0	0.0	0.0	0.0	0.0	0.0	0.0	0.0	0.0	7.0	0.0	0.0	0.0	4.0	0.0	0.0	0.0	0.0	0.0	0.0
Mirtazapine	0.5	1.7		<LOQ	0.0	0.0	0.0	9.4	0.0	75.0	77.0	0.0	0.0	0.0	0.0	<LOQ	0.0	0.0	0.0	21.0	5.3	28.5	216.0	20.0	4.2	20.3	59.0	3.0	2.6	0.0	0.0
Morphine	0.05	0.2		0.3	<LOQ	0.0	0.0	1.5	<LOQ	0.0	0.0	0.0	0.0	0.0	0.0	0.0	0.0	0.0	0.0	2.1	0.4	0.3	1.5	1.2	<LOQ	0.8	0.6	0.0	0.0	0.0	0.0
Nicotine	0.05	0.2		2.2	1.7	5.4	2.8	8.7	3.9	38.5	15.0	4.4	3.2	2.8	0.0	1.9	0.0	8.0	2.2	30.0	3.8	27.5	17.5	18.0	3.0	26.5	20.0	3.1	1.5	1.8	1.6
Niflumic acid	0.5	1.7		0.0	0.0	0.0	0.0	0.0	0.0	0.0	0.0	0.0	0.0	0.0	0.0	0.0	0.0	0.0	0.0	0.0	0.0	0.0	4.3	0.0	0.0	0.0	1.5	0.0	0.0	0.0	0.0
Norfloxacin	0	0.0		n.d.	n.d.	n.d.	n.d.	n.d.	n.d.	n.d.	n.d.	n.d.	n.d.	n.d.	n.d.	n.d.	n.d.	n.d.	n.d.	n.d.	n.d.	n.d.	n.d.	n.d.	n.d.	n.d.	n.d.	n.d.	n.d.	n.d.	n.d.
Octocrylene	0	0.0		n.d.	n.d.	n.d.	n.d.	n.d.	n.d.	n.d.	n.d.	n.d.	n.d.	n.d.	n.d.	n.d.	n.d.	n.d.	n.d.	n.d.	n.d.	n.d.	n.d.	n.d.	n.d.	n.d.	n.d.	n.d.	n.d.	n.d.	n.d.
Ofloxacin	2.5	8.3		n.d.	n.d.	n.d.	n.d.	n.d.	n.d.	n.d.	n.d.	n.d.	n.d.	n.d.	n.d.	n.d.	n.d.	n.d.	n.d.	n.d.	n.d.	n.d.	n.d.	n.d.	n.d.	n.d.	n.d.	n.d.	n.d.	n.d.	n.d.
Omeprazole	5	16.7		0.0	0.0	0.0	0.0	0.0	0.0	30.5	<LOQ	0.0	0.0	0.0	0.0	0.0	0.0	0.0	0.0	0.0	0.0	0.0	19.0	0.0	0.0	0.0	0.0	0.0	0.0	0.0	0.0
Oxazepam	0.4	1.3		2.0	2.0	0.0	2.0	16.2	<LOQ	42.5	78.5	4.0	4.0	4.3	37.5	<LOQ	0.0	1.5	0.0	31.0	<LOQ	87.0	100.5	10.0	6.8	110.5	116.0	8.2	<LOQ	6.0	27.5
Oxycodone	0.5	1.7		0.0	0.0	0.0	0.0	<LOQ	0.0	<LOQ	1.8	0.0	0.0	0.0	0.0	0.0	0.0	0.0	0.0	3.0	0.0	5.5	7.7	<LOQ	0.0	3.0	8.5	0.0	0.0	0.0	0.0
Paroxetine	0.1	0.3		n.d.	n.d.	n.d.	n.d.	n.d.	n.d.	n.d.	n.d.	n.d.	n.d.	n.d.	n.d.	n.d.	n.d.	n.d.	n.d.	n.d.	n.d.	n.d.	n.d.	n.d.	n.d.	n.d.	n.d.	n.d.	n.d.	n.d.	n.d.
perfluorobutane sulfonic acid (PFBS)	0.05	0.2	0.05	0.2	0.2	0.2	0.2	0.2	0.2	0.2	0.2	0.6	0.5	0.5	0.3	0.7	0.3	0.4	0.4	1.8	0.3	0.8	1.8	1.5	0.3	0.7	1.2	1.4	0.4	0.5	0.7
perfluorobutanoic acid (PFBA)	5	16.7	0	n.d.	n.d.	n.d.	n.d.	n.d.	n.d.	n.d.	n.d.	n.d.	n.d.	n.d.	n.d.	n.d.	n.d.	n.d.	n.d.	n.d.	n.d.	n.d.	n.d.	n.d.	n.d.	n.d.	n.d.	n.d.	n.d.	n.d.	n.d.
perfluorodecanoic acid (PFDA)	0.05	0.2	0.08	0.2	0.2	0.2	0.3	0.2	0.2	0.3	0.2	0.2	0.2	0.2	0.1	0.2	0.1	0.3	0.2	0.3	0.2	0.4	1.0	0.3	0.2	0.4	0.6	0.2	0.3	0.2	0.3
perfluorododecanoic acid (PFDoDA)	0.1	0.3	0	<LOQ	0.0	0.0	0.0	0.0	0.0	0.0	0.0	<LOQ	0.2	0.0	0.0	<LOQ	0.0	0.0	0.0	0.0	0.0	0.2	0.0	<LOQ	<LOQ	<LOQ	0.0	0.0	0.0	0.0	0.0
perfluoroheptanoic acid (PFHpA)	0.05	0.2	0.10	<LOQ	0.0	0.0	0.0	<LOQ	0.3	0.0	0.5	0.5	0.5	0.3	0.3	0.5	0.3	0.7	0.6	0.6	0.3	0.6	1.1	0.7	0.4	0.7	0.8	0.5	0.4	0.7	0.7
perfluorohexane sulfonic acid (PFHxS)	0.05	0.2	0.05	0.6	0.5	0.6	0.5	0.7	0.6	0.5	0.6	15.5	8.4	12.4	10.7	5.0	1.0	5.6	5.6	14.0	5.5	17.3	32.4	14.5	5.3	17.5	30.6	10.8	6.8	11.7	10.8
perfluorohexanoic acid (PFHxA)	0.05	0.2	0.25	0.0	0.0	0.0	0.0	0.0	0.0	0.0	0.0	0.0	0.0	0.0	0.0	0.0	0.0	0.0	2.5	2.0	1.4	2.9	5.7	2.6	2.5	3.0	4.8	2.0	2.0	3.2	2.5
perfluorononanoic acid (PFNA)	0.05	0.2	0.05	0.4	0.3	0.5	0.8	0.2	0.2	0.3	0.2	0.2	0.2	0.2	0.2	0.2	0.2	0.3	0.3	0.3	0.2	0.4	1.0	0.3	0.2	0.4	0.5	0.2	0.3	0.5	0.4
perfluorooctane sulfonamide (FOSA)	0.05	0.2	0	0.0	0.0	0.0	0.0	0.0	0.0	0.0	0.0	0.0	0.0	0.0	0.0	0.0	0.0	0.0	0.0	0.0	0.0	0.1	0.2	0.0	0.0	0.1	0.0	0.0	0.0	0.0	0.0
perfluorooctane sulfonic acid (PFOS)	0.05	0.2	0.03	0.9	1.0	1.0	1.0	1.0	1.0	3.4	0.5	6.6	5.8	13.0	5.6	1.8	1.8	3.3	1.9	7.6	2.4	8.7	17.0	2.7	2.6	8.5	14.1	0.0	6.8	7.0	9.1
perfluorooctanoic acid (PFOA)	0.05	0.2	0	0.0	0.0	0.0	0.0	0.0	0.0	0.0	0.0	0.0	0.0	0.0	0.0	0.0	0.0	0.0	0.0	0.0	0.0	0.0	0.0	0.0	0.0	0.0	0.0	0.0	0.0	0.0	0.0
perfluoropentanoic acid (PFPeA)	5	16.7	0	n.d.	n.d.	n.d.	n.d.	n.d.	n.d.	n.d.	n.d.	n.d.	n.d.	n.d.	n.d.	n.d.	n.d.	n.d.	n.d.	n.d.	n.d.	n.d.	n.d.	n.d.	n.d.	n.d.	n.Pe.	n.d.	n.d.	n.d.	n.d.
perfluorotetradecanoic acid (PFTeDA)	0.05	0.2	0	n.d.	n.d.	n.d.	n.d.	n.d.	n.d.	n.d.	n.d.	n.d.	n.d.	n.d.	n.d.	n.d.	n.d.	n.d.	n.d.	n.d.	n.d.	n.d.	n.d.	n.d.	n.d.	n.d.	n.d.	n.d.	n.d.	n.d.	n.d.
perfluoroundecanoic acid (PFUnDA)	0.05	0.2	0	0.3	<LOQ	<LOQ	0.5	<LOQ	0.0	<LOQ	0.0	0.0	0.0	0.0	0.0	0.2	0.2	<LOQ	0.0	0.2	0.2	0.2	0.2	0.2	0.2	0.2	0.2	0.2	0.2	0.0	0.0
Propanolol	0.25	0.8		0.0	0.0	0.0	0.0	<LOQ	0.0	1.3	1.1	0.0	0.0	0.0	0.0	0.0	0.0	<LOQ	0.0	4.0	1.0	4.1	7.5	12.0	1.5	3.0	3.0	0.0	0.0	0.0	0.0
Propylparaben	0.25	0.8		0.0	0.0	0.0	0.0	0.0	0.0	0.0	0.0	0.0	0.0	0.0	0.0	0.0	0.0	0.0	0.0	1.9	0.0	0.0	0.0	6.0	0.0	0.0	0.0	1.0	0.0	0.0	0.0
Ranitidine	0.5	1.7		0.0	0.0	0.0	0.0	0.0	0.0	3.0	4.1	0.0	0.0	0.0	0.0	0.0	0.0	0.0	0.0	<LOQ	0.0	1.5	8.0	0.0	0.0	1.4	0.0	0.0	0.0	0.0	0.0
Roxithromycin	0	0.0		n.d.	n.d.	n.d.	n.d.	n.d.	n.d.	n.d.	n.d.	n.d.	n.d.	n.d.	n.d.	n.d.	n.d.	n.d.	n.d.	n.d.	n.d.	n.d.	n.d.	n.d.	n.d.	n.d.	n.d.	n.d.	n.d.	n.d.	n.d.
Sertraline	0	0.0		n.d.	n.d.	n.d.	n.d.	n.d.	n.d.	n.d.	n.d.	n.d.	n.d.	n.d.	n.d.	n.d.	n.d.	n.d.	n.d.	n.d.	n.d.	n.d.	n.d.	n.d.	n.d.	n.d.	n.d.	n.d.	n.d.	n.d.	n.d.
Sotalol	0.15	0.5		3.2	<LOQ	0.0	0.0	4.0	1.6	4.5	11.0	<LOQ	0.0	0.0	0.0	0.0	0.0	0.0	0.0	18.0	3.2	14.0	11.0	16.0	2.1	8.0	10.5	2.5	2.2	1.4	1.6
Sucralose	5	16.7		22.0	<LOQ	<LOQ	35.5	91.0	<LOQ	159.0	240.0	45.5	35.0	60.0	65.5	38.0	0.0	49.5	44.0	311.0	98.5	453.5	1394.5	315.5	77.5	395.0	600.0	208.5	110.0	150.5	238.5
Sulfamethoxazole	1.5	5.0		0.0	0.0	0.0	0.0	0.0	0.0	0.0	0.0	0.0	0.0	0.0	0.0	0.0	0.0	0.0	0.0	34.0	0.0	0.0	0.0	13.0	0.0	0.0	0.0	6.4	0.0	0.0	0.0
Terbutryn	1	3.3		n.d.	n.d.	n.d.	n.d.	n.d.	n.d.	n.d.	n.d.	n.d.	n.d.	n.d.	n.d.	n.d.	n.d.	n.d.	n.d.	n.d.	n.d.	n.d.	n.d.	n.d.	n.d.	n.d.	n.d.	n.d.	n.d.	n.d.	n.d.
tetracycline	0	0.0		n.d.	n.d.	n.d.	n.d.	n.d.	n.d.	n.d.	n.d.	n.d.	n.d.	n.d.	n.d.	n.d.	n.d.	n.d.	n.d.	n.d.	n.d.	n.d.	n.d.	n.d.	n.d.	n.d.	n.d.	n.d.	n.d.	n.d.	n.d.
Tramadol	0.25	0.8		<LOQ	0.0	<LOQ	1.1	30.0	<LOQ	10.6	32.8	1.8	0.0	0.0	0.8	4.0	0.0	2.3	0.0	114.0	3.7	19.0	47.0	40.0	2.8	11.5	28.9	7.6	5.7	4.8	4.0
Trimethoprim	0.1	0.3		0.0	0.0	0.0	0.0	0.0	0.0	0.0	8.5	0.0	0.0	0.0	0.0	0.0	0.0	0.0	0.0	4.0	1.5	6.2	20.0	4.0	0.5	6.2	58.0	<LOQ	<LOQ	0.5	0.5
Valproic acid	50	166.7		n.d.	n.d.	n.d.	n.d.	n.d.	n.d.	n.d.	n.d.	n.d.	n.d.	n.d.	n.d.	n.d.	n.d.	n.d.	n.d.	n.d.	n.d.	n.d.	n.d.	n.d.	n.d.	n.d.	n.d.	n.d.	n.d.	n.d.	n.d.
Valsartan	7.5	25.0		0.0	0.0	0.0	0.0	74.5	0.0	136.0	222.5	0.0	0.0	0.0	0.0	0.0	0.0	0.0	0.0	45.0	0.0	27.5	59.0	0.0	0.0	0.0	<LOQ	0.0	0.0	0.0	0.0
Venlafaxine	0.3	1.0		0.0	0.0	0.0	0.0	5.0	3.0	17.8	60.5	0.0	<LOQ	<LOQ	1.8	<LOQ	<LOQ	<LOQ	<LOQ	86.0	10.0	156.0	493.5	42.0	10.2	87.0	178.5	11.0	2.0	12.4	9.5
**TOTAL ng/Sampler**				158	108	223	156	1119	228	1748	2666	322	220	205	321	178	117	204	181	1731	473	2304	6100	1270	335	1660	2917	425	264	417	488

n.d. = not detected
